# Supplementary material for: Nanoparticle-based hollow microstructures formed by two-stage nematic nucleation and phase separation
Source: Nat Commun. 2019 Feb 22;10:894. doi: 10.1038/s41467-019-08702-3 (PMC6385213; doi:10.1038/s41467-019-08702-3)
Supplement: Supplementary file 1 — Supplementary Information [file 41467_2019_8702_MOESM1_ESM.pdf]

Supplementary Information

Nanoparticle-Based Hollow Microstructures Formed by Two-Stage Nematic Nucleation and Phase Separation

Riahinasab et al.

Concentration/Cooling rate	7 °C/min	15 °C/min	20 °C/min	30 °C/min	200 °C/min
0.075 wt %	30 µm (n=12)	27 µm (n=72)	22 µm (n=49)	15 µm (n=183)	4 µm (n=68)
0.15 wt %	81 µm (n=10)	45 µm (n=57)	35 µm (n=78)	23 µm (n=137)	10 µm (n=130)
0.3 wt %	96 µm (n=10)	64 µm (n=45)	51 µm (n=55)	38 µm (n=70)	19 µm (n=47)

Supplementary Table 1. Average structure diameter measurements for the assembled structures as plotted in Figure 2h and Figure 2i. For each data point, the total number of measured structures, n, ranged from 10–183.

Supplementary Note I. Fluorescence Microscopy Time Sequence of Single Compartment Capsule Formation

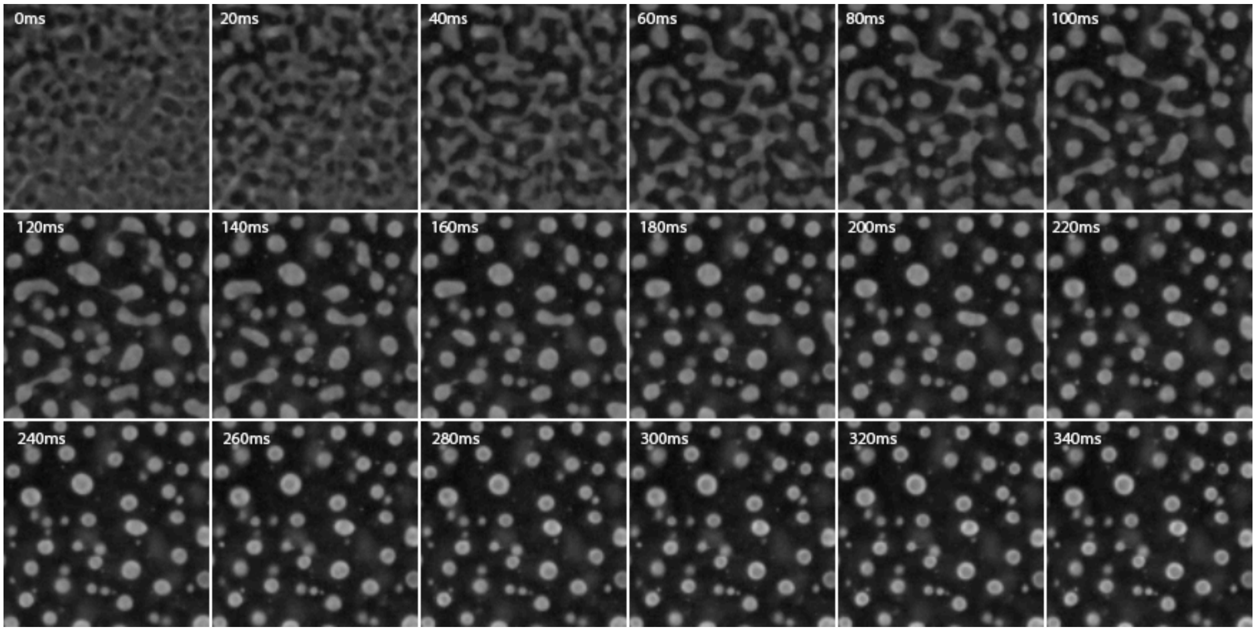

Supplementary Figure 1. Fluorescence microscopy time sequence of single compartment capsule formation recorded with high speed video capture. Snapshots were taken every 20 ms.

Supplementary Note II. Concentration-Dependence of Isotropic-to-Nematic Phase Transition Temperature

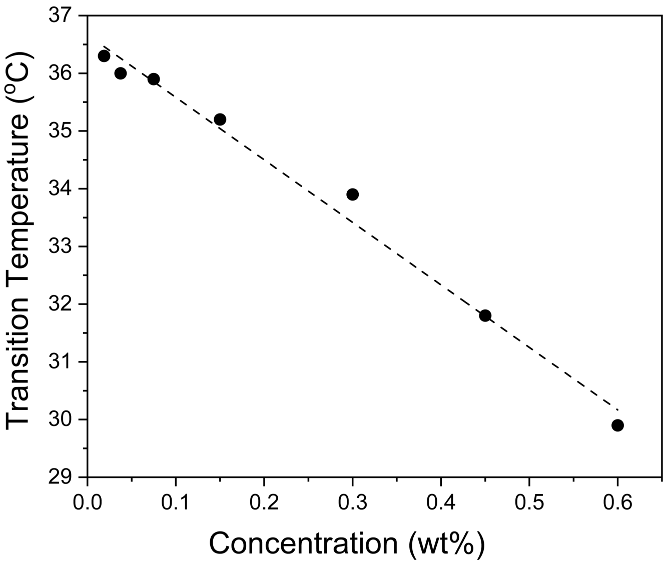

Supplementary Figure 2. Liquid crystal isotropic-to-nematic phase transition temperature as a function of nanoparticle concentration.

Functionalized quantum dot nanoparticles (in toluene) were dispersed in liquid crystal (5CB) at seven different concentrations (x-axis = 0.01875 wt %, 0.0375 wt %, 0.075 wt %, 0.15 wt %, 0.3 wt %, 0.45 wt %, 0.6 wt %) and then sonicated for two hours to obtain a uniform distribution and evaporate any residual solvent. The mixtures were maintained in the isotropic phase in an oven, then 0.5 μl of each was pipetted onto a cleaned glass slide and then covered with a coverslip. Slides were transferred onto the surface of a heating stage held at 40 °C and cooled at rate of 30 °C/min. The isotropic-to-nematic phase transition temperature was recorded at the onset of birefringence using a polarized optical microscope.

Supplementary Note III. General Considerations for Ligand Synthesis

Reactants, reagents, and solvents were used as received from commercial suppliers unless otherwise noted. A Mettler-Toledo XS105 balance (repeatable to 0.1 mg with minimum 2.0 mg load) was used to measure mass. Flash column chromatography was performed using 40–63 µm 60 Å silica. Thin layer chromatography (TLC) was performed using Silcycle glass-backed extra hard layer indicating plates with 60 Å pore size and thickness of 250 µm, which were stored in a desiccator when not in use. Melting points were obtained on an electrothermal melting point apparatus and are uncorrected. NMR spectra were obtained on Agilent spectrometers. ^{1}H NMR spectra were obtained at 400 MHz and referenced to the residual $CHCl_3$ singlet at 7.26 ppm unless otherwise noted. The abbreviations s, d, t, q, p, dd, and m stand for the resonance multiplicities singlet, doublet, triplet, quartet, pentet, doublet of doublet, and multiplet, respectively. ^{13}C NMR spectra were obtained at 100 MHz and referenced to the center line of the $CDCl_3$ triplet at 77.2 ppm unless otherwise noted. Carbon atom degree of substitution was determined using ^{1}H–^{13}C HSQC.ATR FT-IR analysis was performed on a Bruker Vertex 70 (DOD grant68959-RT-REP). HRMS data were obtained on a Thermo Scientific Exactive Plus Orbitrap mass spectrometer using ESI (DOD grant68959-RT-REP). Glassware for all reactions was oven-dried at 135 °C and cooled in a desiccator prior to use.

Supplementary Note IV. Preparation of the Mesogenic Ligand

A. Overview of the Synthetic Sequence

The mesogenic ligand **8** was obtained following an eight step (five steps longest-linear) sequence as shown below. This sequence was adapted from prior reports.[1,2]

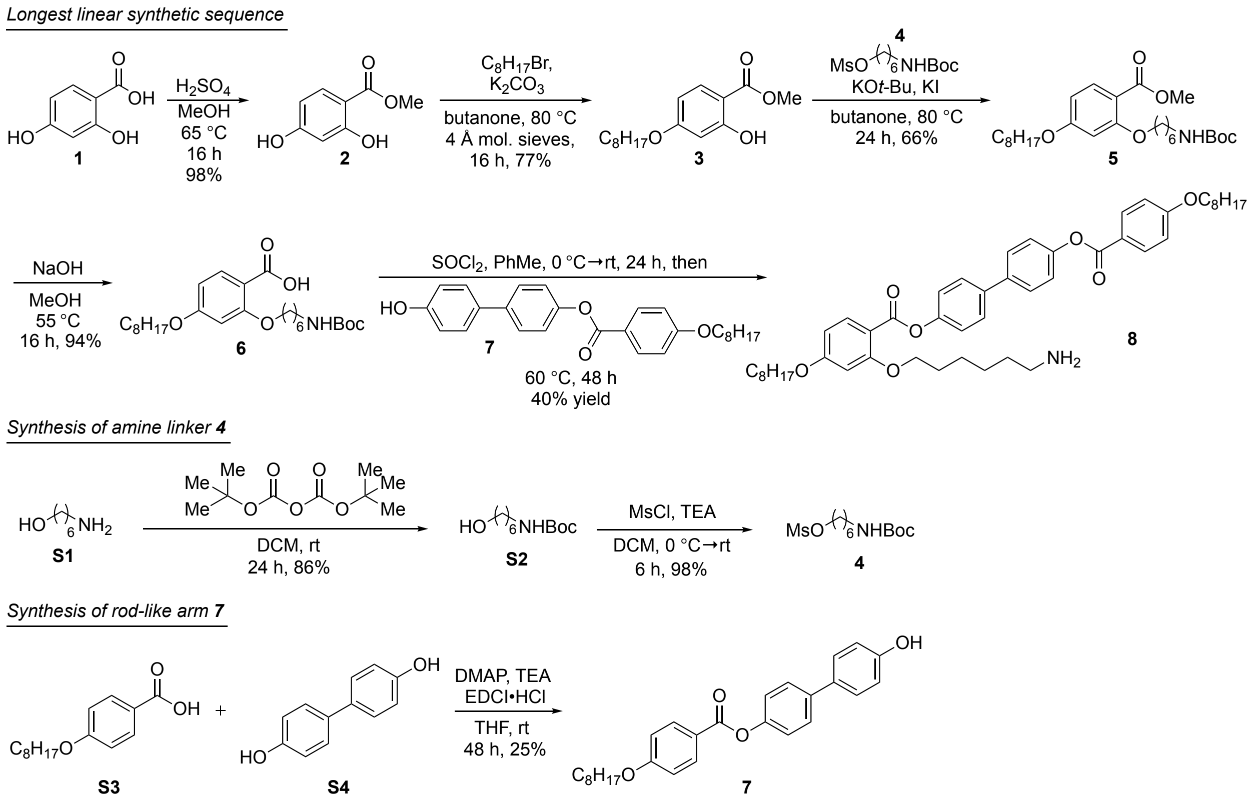

Supplementary Figure 3. Synthesis of the mesogenic ligand.

B. Procedures and Characterization of Individual Steps

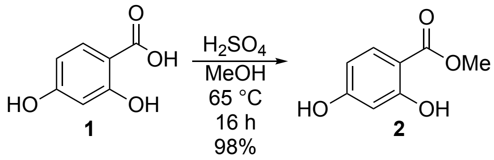

Methyl 2,4-dihydroxybenzoate (2). To a 100 mL round bottom flask equipped with a PTFE-coated magnetic stir barwereadded 30 mL of methanol and 6.00 g (39.0 mmol) of 2,4-dihydroxybenzoic acid **1**. The reaction mixture was placed in an ice bath, then 5.0 mL of concentrated sulfuric acid was added slowly. The reaction flask was heated to reflux for 16 hours. After cooling to ambient temperature, the solvent was removed under vacuum and the residue was poured into 100 mL of ice water. Analytically pure methyl 2,4-dihydroxybenzoate **2** (6.40 g, 98%) was isolated upon filtration. This product is also available commercially.

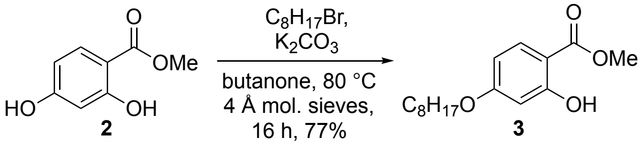

Methyl 2-hydroxy-4-(octyloxy)benzoate (3). To a 250 mL round bottom flask equipped with a PTFE-coated magnetic stir bar, 10 g of 4 Å molecular sieves, and 80 mL of butanone were added 3.9 g (23.2 mmol) of methyl 2,4-dihydroxybenzoate **2** and 16.0 g (116 mmol) of potassium carbonate. The reaction flask was heated to reflux followed by the slow addition of 4.93 g of 1-bromooctane (25.5 mmol) in 30 mL of butanone over a period of one hour. After 16 hours, the reaction was cooled to room temperature and the solids were filtered off. The solution was concentrated under vacuum and the residue was purified by flash column chromatography on SiO_2(100:0→0:100 hexanes:toluene) to afford **3** (4.99 g, 77%) as a white solid, mp = 38°C. The spectral data matched those reported by Hirst and coworkers.[3] ^{1}H NMR (400 MHz, $CDCl_3$): δ 10.95 (s, 1H), 7.72 (d, J = 9.6 Hz, 1H), 6.43 (s, 1H), 6.43–6.40 (m, 1H), 3.96 (t, J = 6.7 Hz, 2H), 3.91 (s, 3H), 1.78 (p, J = 8.2 Hz, 2H), 1.47–1.39 (m, 2H), 1.35–1.24 (m, 8H), 0.88 (t, J = 7.0 Hz, 3H); ^{13}C NMR(100 MHz, $CDCl_3$): δ 170.4 (CO), 165.2 (C), 163.7 (C), 131.1 (CH), 107.9 (CH), 105.2 (C), 101.1 (CH), 68.3 (CH_2), 51.9 (CH_3), 31.8 (CH_2), 29.3 (CH_2), 29.2 (CH_2), 29.0 (CH_2), 25.9 (CH_2), 22.6 (CH_2), 14.1 (CH_3). ATR-FTIR (thin film): 3207, 2923, 2850, 1674, 1618, 1577, 1444, 1332, 1247, 1181 cm^{-1}.

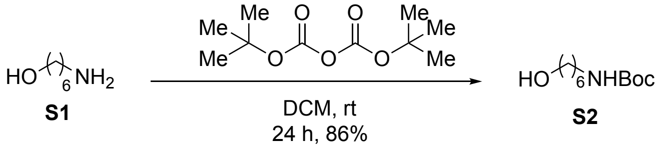

6-((*tert*-Butoxycarbonyl)amino)hexan-1-ol (S2).Into a 150 mL round bottom flask charged with a PTFE-coated magnetic stir bar were added 11 mL of dry DCM and 2.00 g (5.00 mmol) of 6-aminohexan-1-ol **S1**. A solution of di-*tert*-butyl dicarbonate (4.00 g, 18.3 mmol) in 11 mL of dry DCM was then added dropwise to the reaction flask. After 24 hours, the solvent was removed under reduced pressure and the residue was extracted with dilute acetic acid and diethyl ether to afford **S2** (3.42 g, 86%), which was used without purification. The spectral data matched those reported by Hirst and coworkers.[3]

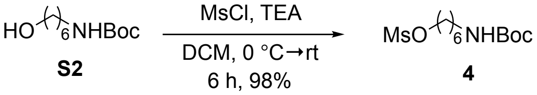

6-((*tert*-Butoxycarbonyl)amino)hexyl methanesulfonate (4). Into a 250 mL round bottom flask charged with a PTFE-coated magnetic stir bar were added 47 mL of dry DCM, 2.00 g (9.4 mmol) of **S2**, and 1.7 mL (12.2 mmol) of triethylamine. The reaction flask was placed in an ice bath and 0.9 mL (11.3 mmol) of methanesulfonyl chloride was added dropwise. After 6 hours, the reaction was quenched with 50 mL of water and separated, and the organic layer was dried over

anhydrous sodium sulfate and removed under reduced pressure to afford **4** (2.72 g, 98%) as alight yellow solid. The spectral data matched those reported by Hirst and coworkers.[3]

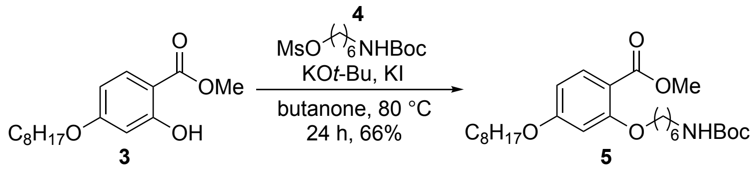

Methyl 2-((6-((*tert*-butoxycarbonyl)amino)hexyl)oxy)-4-(octyloxy)benzoate (5). To a 150 mL round bottom flask charged with a PTFE-coated magnetic stir bar were added 45 mL of butanone, 1.20 g (4.28 mmol) of methyl 2-hydroxy-4-(octyloxy)benzoate**3** and 1.39 g (4.71 mmol) of **4**. Then, 1.07 g (6.42 mmol) of potassium iodide and 0.58 g (5.14 mmol) of potassium tertbutoxide powder were added to the reaction vessel and heated to reflux for 24 hours. The solvent was then removed under reduced pressure and the residue was extracted with DCM and water. The organic layer was collected and dried over anhydrous sodium sulfate before it was concentrated under vacuum. Purification by flash column chromatography (100:0→90:10 chloroform:methanol) on SiO_2 afforded **5** (1.35 g, 66%) as a light yellow oil. The spectral data matched those reported by Hirst and coworkers.[3] ^1H NMR (400 MHz, $CDCl_3$): δ 7.82 (d, J = 8.6 Hz, 1H), 6.68–6.43 (m, 2H), 4.57 (br s, 1H), 3.98 (q, J = 6.4 Hz, 4H), 3.84 (s, 3H), 3.16–3.06 (m, 2H), 1.89–1.72 (m, 4H), 1.53–1.45 (m, 5H), 1.43 (s, 9H), 1.37–1.22 (m, 11H), 0.88 (t, J = 7.0, 3H); ^{13}C NMR (100 MHz, $CDCl_3$): δ 166.3 (C), 163.7 (C), 160.8 (C), 156.0 (C), 133.8 (CH), 112.2 (C), 105.1 (CH), 100.3 (CH), 77.2 (C), 68.7 (CH_2), 68.2 (CH_2), 51.6 (CH_3), 40.5 (CH_2), 31.8 (CH_2), 29.3 (CH_2), 29.2 (CH_2), 29.1 (CH_2), 29.0 (CH_2), 28.8 (CH_2), 28.4 ($3CH_3$), 26.4 (CH_2), 26.0 (CH_2), 25.7 (CH_2), 22.6 (CH_2), 14.1 (CH_3). ATR-FTIR (neat): 3375, 2927, 2856, 1704, 1608, 1506, 1250, 1175 cm^{-1}.

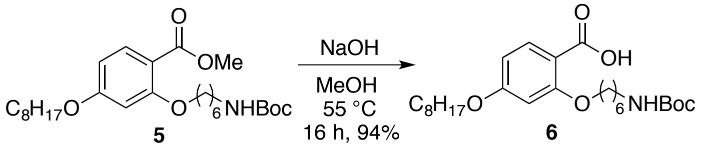

2-((6-((*tert*-butoxycarbonyl)amino)hexyl)oxy)-4-(octyloxy)benzoic acid (6).To a 150 mL round bottom flask charged with a PTFE-coated magnetic stir barwere added 57 mL of methanol and1.32 g (2.75 mmol) of **5**. Then, a solution of 1.13 g of NaOH in 14 mL of deionized water was added slowly to the flask. The reaction was stirred at 55 °C for 16 hours.After cooling to room temperature, the solvent was removed under reduced pressure and the aqueous residue was acidified with dilute HCl prior to extraction with DCM. The organic layer was dried over anhydrous sodium sulfate before it was concentrated under vacuum.Purification by flash column chromatography (100:0→90:10 chloroform:methanol)afforded **6** (1.20 g, 94%) as a white solid, mp= 86 °C. The spectral data matched those reported by Hirst and coworkers.[3] ^1H NMR (400 MHz, $CDCl_3$): δ 10.72 (br s, 1H), 8.10 (d, J = 8.7 Hz, 1H), 6.61 (dd, J = 8.8, 2.3 Hz, 1H), 6.48 (d, J = 2.3 Hz, 1H), 4.53 (br s, 1H), 4.19 (t, J = 6.7 Hz, 2H), 4.00 (t, J = 6.7 Hz, 2H), 3.16–3.07 (m, 2H), 1.91 (p, J = 7.8 Hz,2H), 1.79 (p, J = 8.2 Hz,2H), 1.56–1.45 (m, 5H), 1.43 (s, 9H), 1.42–1.24

(m, 11H), 0.88 (t, J = 6.6, 3H); ^{13}C NMR (100 MHz, $CDCl_3$): δ165.3 (C), 164.6 (C), 158.9 (C), 156.0 (C), 135.4 (CH), 110.2 (C), 107.1 (CH), 99.8 (CH), 77.2 (C), 70.0 (CH_2), 68.6 (CH_2), 40.3 (CH_2), 31.8 (CH_2), 29.9 (CH_2), 29.3 (CH_2), 29.2 (CH_2), 29.0 (CH_2), 28.8 (CH_2), 28.4 ($3CH_3$), 26.3 (CH_2), 25.9 (CH_2), 25.6 (CH_2), 22.6 (CH_2), 14.1 (CH_3). ATR-FTIR (neat): 3303, 2927, 1608, 1533, 1439, 1267, 1197, 1126 cm^{-1}. HRMS (ESI) *m/z* calculated for $C_{26}H_{43}NO_6$ $[M]^+$: 466.3163, found: 466.3117.

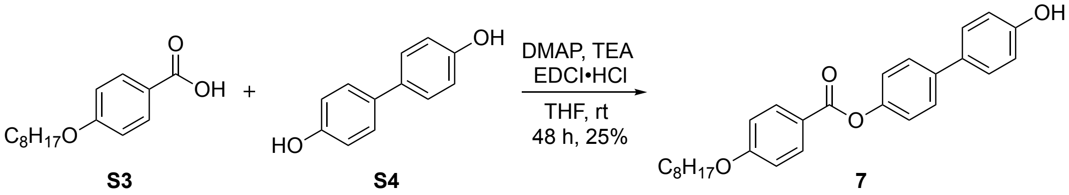

4'-Hydroxy-[1,1'-biphenyl]-4-yl 4-(octyloxy)benzoate (7). To a 100 mL round bottom flask charged with a PTFE-coated magnetic stir bar were added 44 mL of THF, 2.00 g (8.00 mmol)of 4-(octyloxy)benzoic acid **S3**, 0.20 g (1.63 mmol) of DMAP, and 1.49 g (8.00 mmol) of **S4**. Then, 2.6 mL (18.4 mmol) of triethylamine was added to the reaction flask, followed by 1.84 g (9.60 mmol) of EDCI•HCl.The reaction was allowed to stir for 48 hours at room temperature. The solids were then filtered and washed with a minimal amount of cold DCM. The solvent was removed under vacuum and the residue was treated with ethanol and heated to reflux and filtered immediately once hot. The filtrate was cooled to room temperature and filtered again. The solid residues were collected and purified by flash column chromatography (100:0→80:20 hexanes:EtOAc) to afford **7** (0.84 g, 25%) as a white solid, mp = 168 °C. ^{1}H NMR (400 MHz, $CDCl_3$): δ 8.16 (d, J = 8.8 Hz, 2H), 7.56 (d, J = 8.7 Hz, 2H), 7.45 (d, J = 8.7 Hz, 2H), 7.24 (d, J = 8.7 Hz, 2H), 6.98 (d, J = 8.7 Hz, 2H), 6.89 (d, J = 8.7 Hz, 2H), 4.05 (t, J = 6.8 Hz, 2H), 1.83 (p, J = 6.8 Hz, 2H), 1.48 (p, J = 6.8 Hz, 2H), 1.38–1.26 (m, 8H), 0.89 (t, J = 6.7 Hz, 3H): ^{13}C NMR (100 MHz, $CDCl_3$): δ 165.1 (C), 163.5 (C), 155.1 (C), 150.0 (C), 138.4 (C), 133.2 (C), 132.3 (2CH),128.4 (2CH), 127.7 (2CH), 122.0 (2CH), 121.5 (C), 115.6 (2CH), 114.3 (2CH), 68.3 (CH_2), 31.8 (CH_2), 29.3 (CH_2), 29.2 (CH_2), 29.1 (CH_2), 26.0 (CH_2), 22.6 (CH_2), 14.1 (CH_3). ATR-FTIR (neat): 3458, 2920, 2853, 1748, 1606, 1497, 1254, 1166 cm^{-1}.

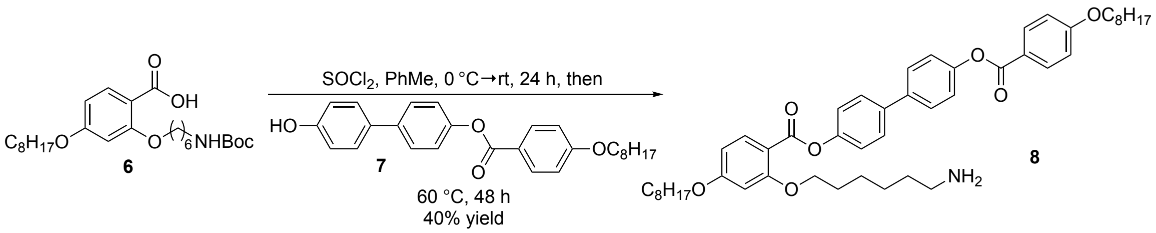

4'-((4-(Octyloxy)benzoyl)oxy)-[1,1'-biphenyl]-4-yl 2-((6-aminohexyl)oxy)-4-(octyloxy)benzoate (8). To a 25 mL round bottom flask charged with a PTFE-coated magnetic stir bar were added 7.6 mL of anhydrous toluene and 0.64 g of **6**(1.37 mmol). Then, 0.2 mL of thionyl chloride (2.5 mmol) was added dropwise at 0 °C and the reaction was allowed to warm to room temperature and stir for 24 hours. Finally, 0.45 g (1.07 mmol) of **7** was added to the flask and the reaction was heated to 60 °C for 48 hours. After cooling to room temperature and concentration under vacuum, purification of the residue by flash column chromatography (80:20:00hexanes:ethylacetate:methanol→00:50:50 hexanes:ethylacetate:methanol on Et_3N-treated SiO_2) afforded **8** (0.330 g, 40%) as a white solid, R_f = 0.89 (50:50EtOAc:MeOH on an Et_3N-treated SiO_2 TLC plate, visualized by 254 nm light), mp = 94 °C.^{1}H NMR (400 MHz, $CDCl_3$): δ 8.16 (d, J = 8.8 Hz, 2H), 8.05 (d, J = 9.1 Hz, 1H), 7.61 (d, J = 8.6 Hz, 2H), 7.60 (d, J = 8.4 Hz, 2H), 7.27 (d, J = 8.6 Hz, 2H), 7.26 (d, J = 8.4 Hz, 2H), 6.98 (d, J = 8.7 Hz, 2H), 6.53 (dd, J = 9.0, 6.5 Hz, 1H), 6.49 (d, J = 2.5 Hz, 1H), 4.05 (t, J = 6.5 Hz, 2H), 4.02 (t, J = 6.5 Hz, 2H), 4.01 (t, J = 6.5 Hz, 2H), 2.81 (br s, 2H), 2.67 (t, J = 6.4 Hz, 2H), 1.91–1.77 (m, 6H), 1.57–1.43 (m, 9H), 1.41–1.26 (m, 17H),0.91 (t, J = 6.5 Hz, 3H), 0.90 (t, J = 6.5 Hz, 3H); ^{13}C NMR (125 MHz, $CDCl_3$): δ 167.9(C),167.3 (C), 166.8 (C), 166.3 (C), 164.5 (C), 153.2 (C), 153.1 (C), 140.7 (C), 140.3 (C), 137.1 (CH), 135.0 (2CH), 130.9 (4CH), 125.0 (2CH), 124.8 (2CH), 124.0 (C), 117.0 (2CH), 113.3 (C), 108.2 (CH), 102.8 (CH), 71.4 (CH_2), 71.0 ($2CH_2$), 42.6 (CH_2), 34.5 (CH_2), 32.0 ($2CH_2$), 31.9 ($2CH_2$), 31.8 (CH_2), 31.7 (CH_2), 31.3 (CH_2), 29.7 (CH_2), 28.7 ($2CH_2$), 28.6 (CH_2), 28.0 (CH_2), 26.8 (CH_2), 25.3 ($2CH_2$), 16.8 ($2CH_3$). ATR-FTIR (neat): 2923, 2854, 1726, 1605, 1251, 1196, 1162 cm^{-1}. HRMS (ESI) m/z calculated for $C_{48}H_{63}NO_7$ $[M]^+$: 766.4677, found: 766.4659.

Supplementary Note V. Ligand Exchange and Quantification by ^{1}H NMR Spectroscopy

Ligand exchange was executed and quantified by ^{1}H NMR following our published procedure.[3]

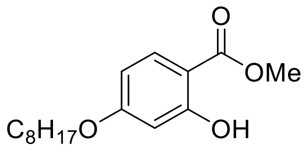

3

^1H (400 MHz), $CDCl_3$

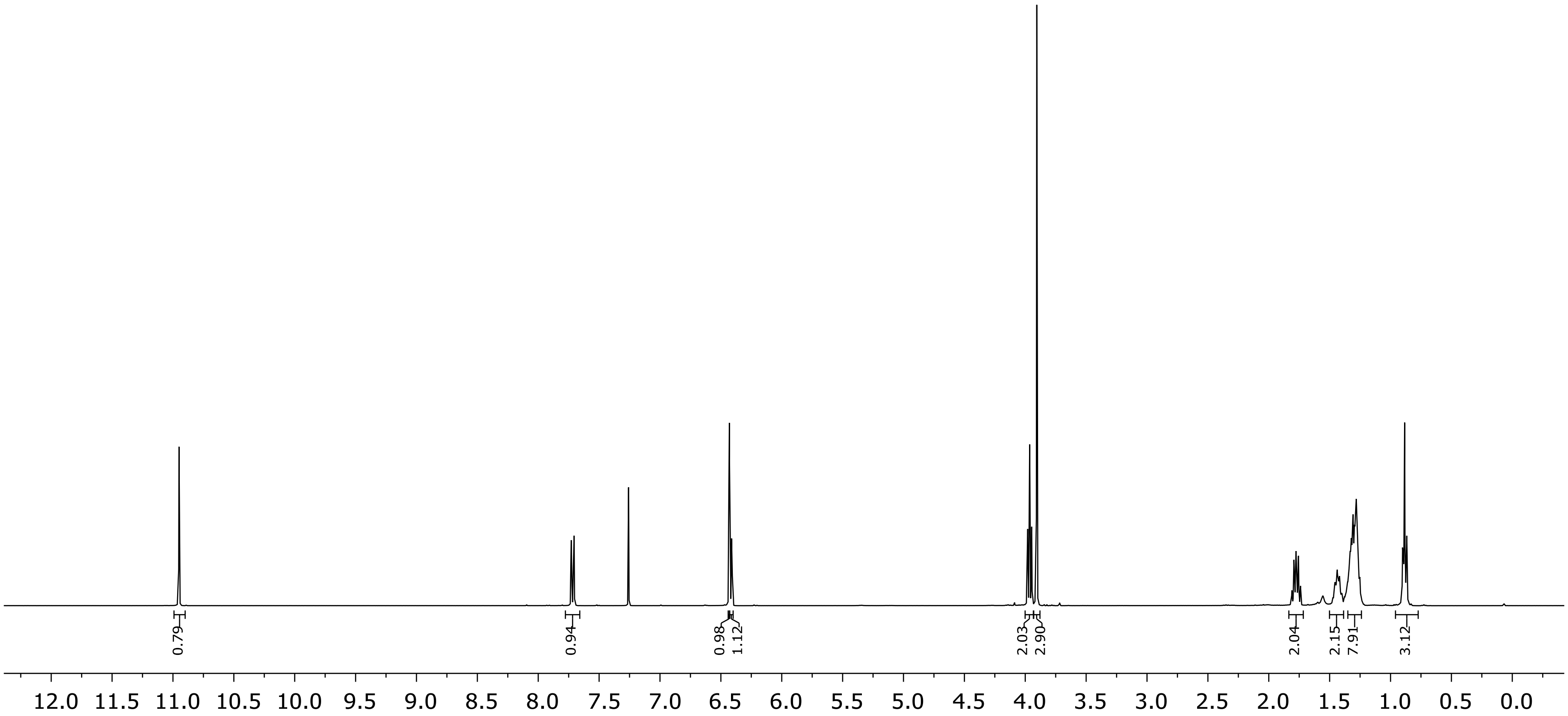

Supplementary Figure 4. ^{1}H NMR of **3**.

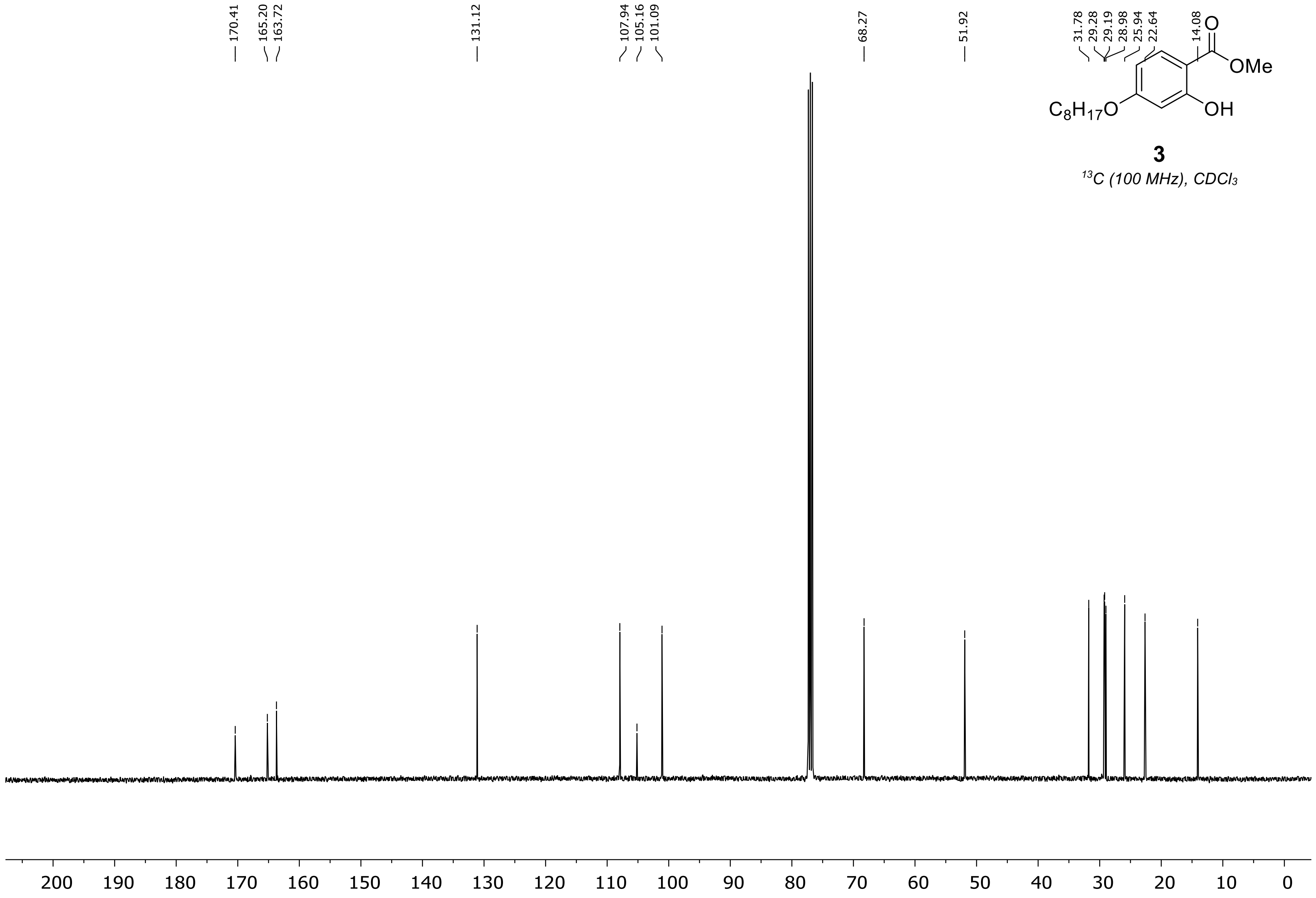

Supplementary Figure 5. ^{13}C NMR of **3**.

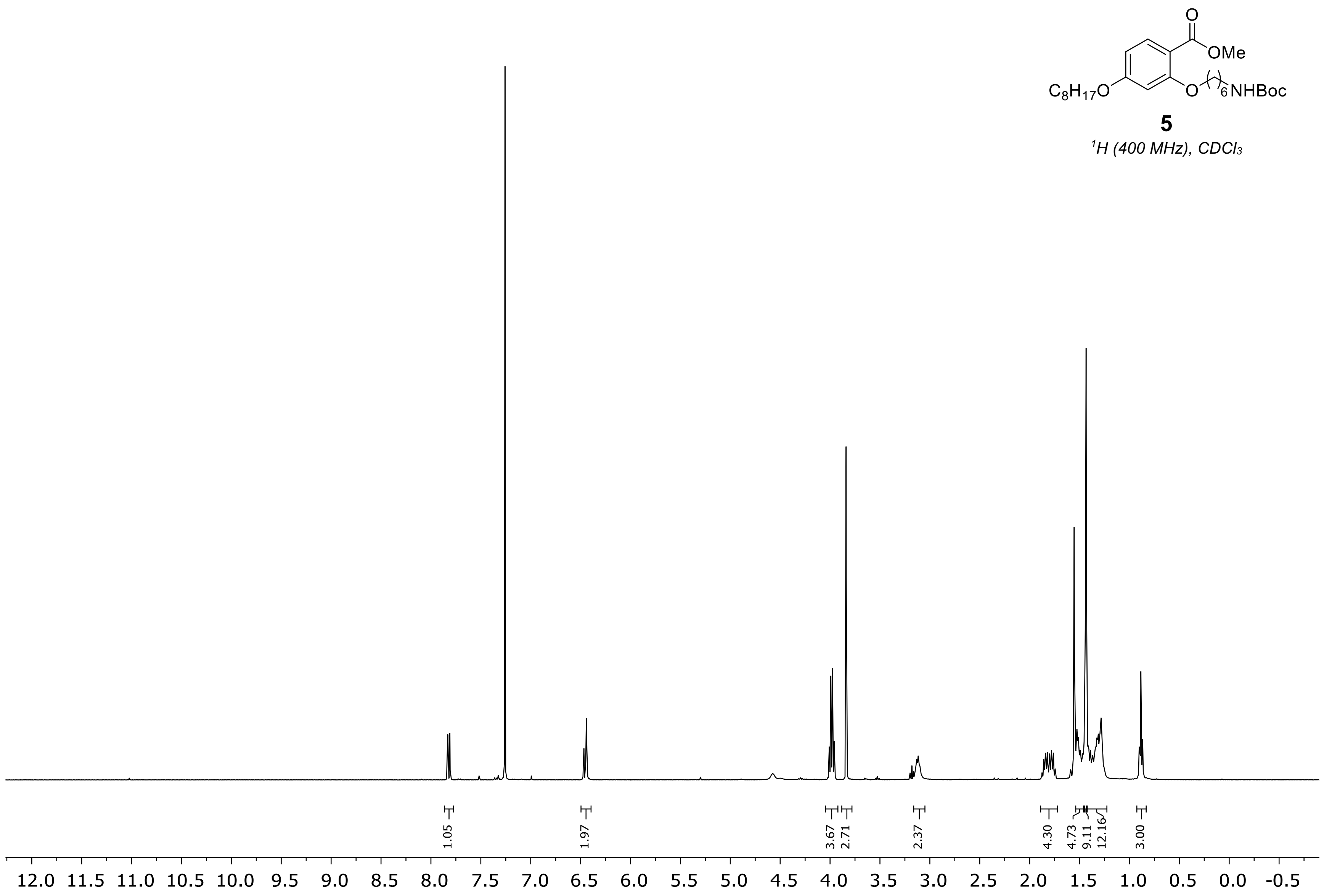

Supplementary Figure 6. ^{1}H NMR of **5**.

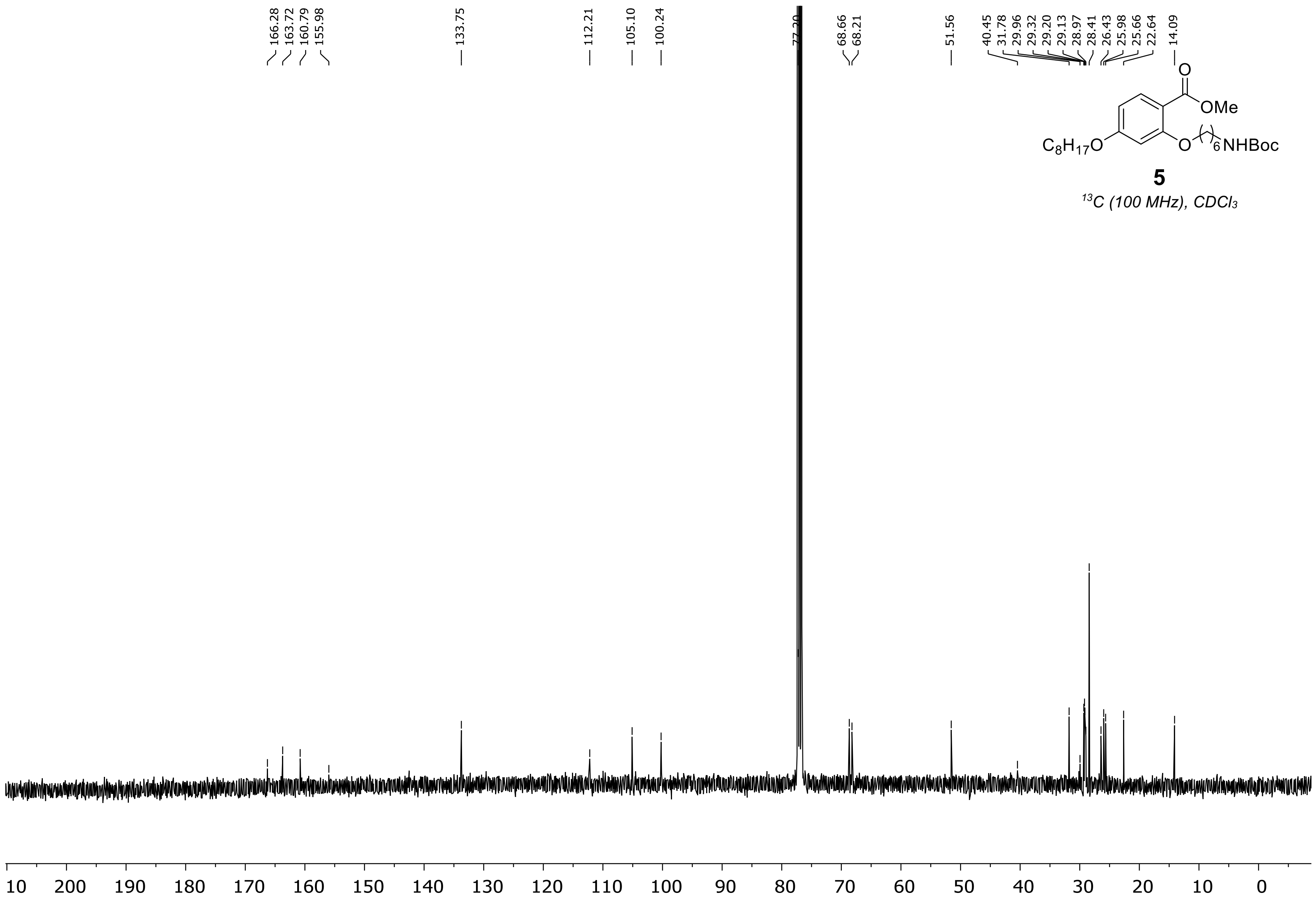

Supplementary Figure 7. ^{13}C NMR of **5**.

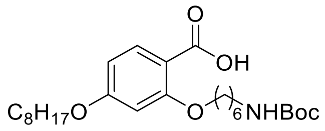

6

¹H (400 MHz), CDCl₃

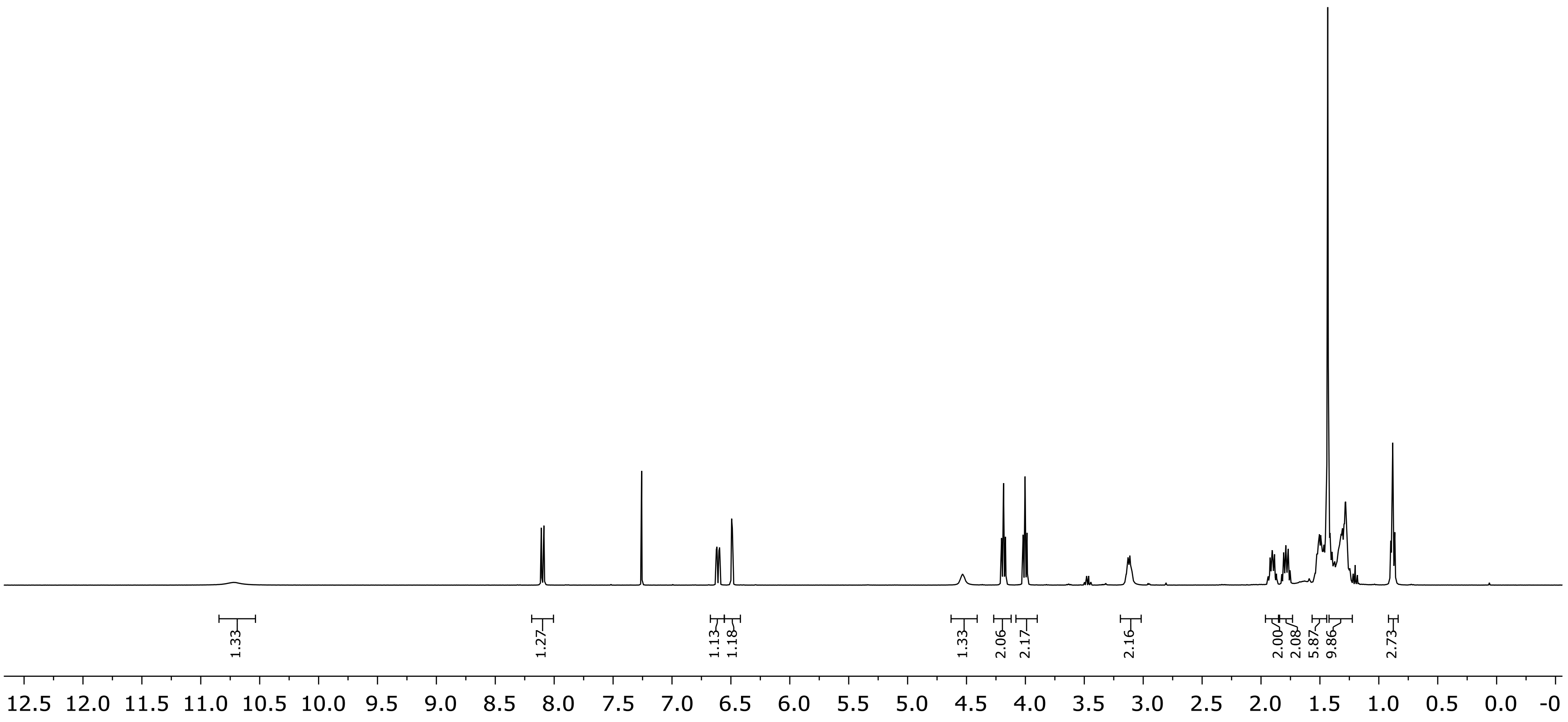

Supplementary Figure 8. ^1H NMR of **6**.

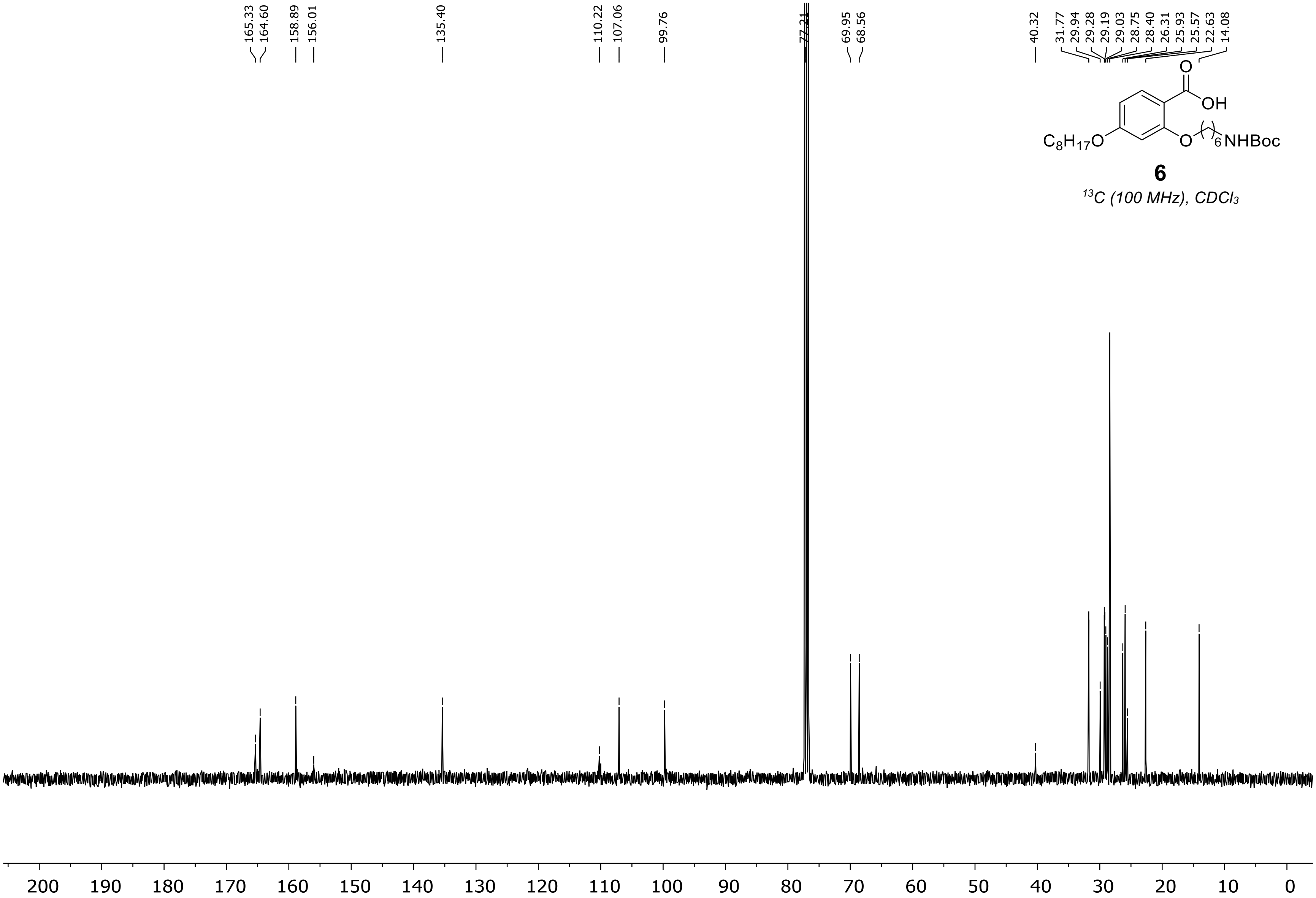

Supplementary Figure 9. ^{13}C NMR of **6**.

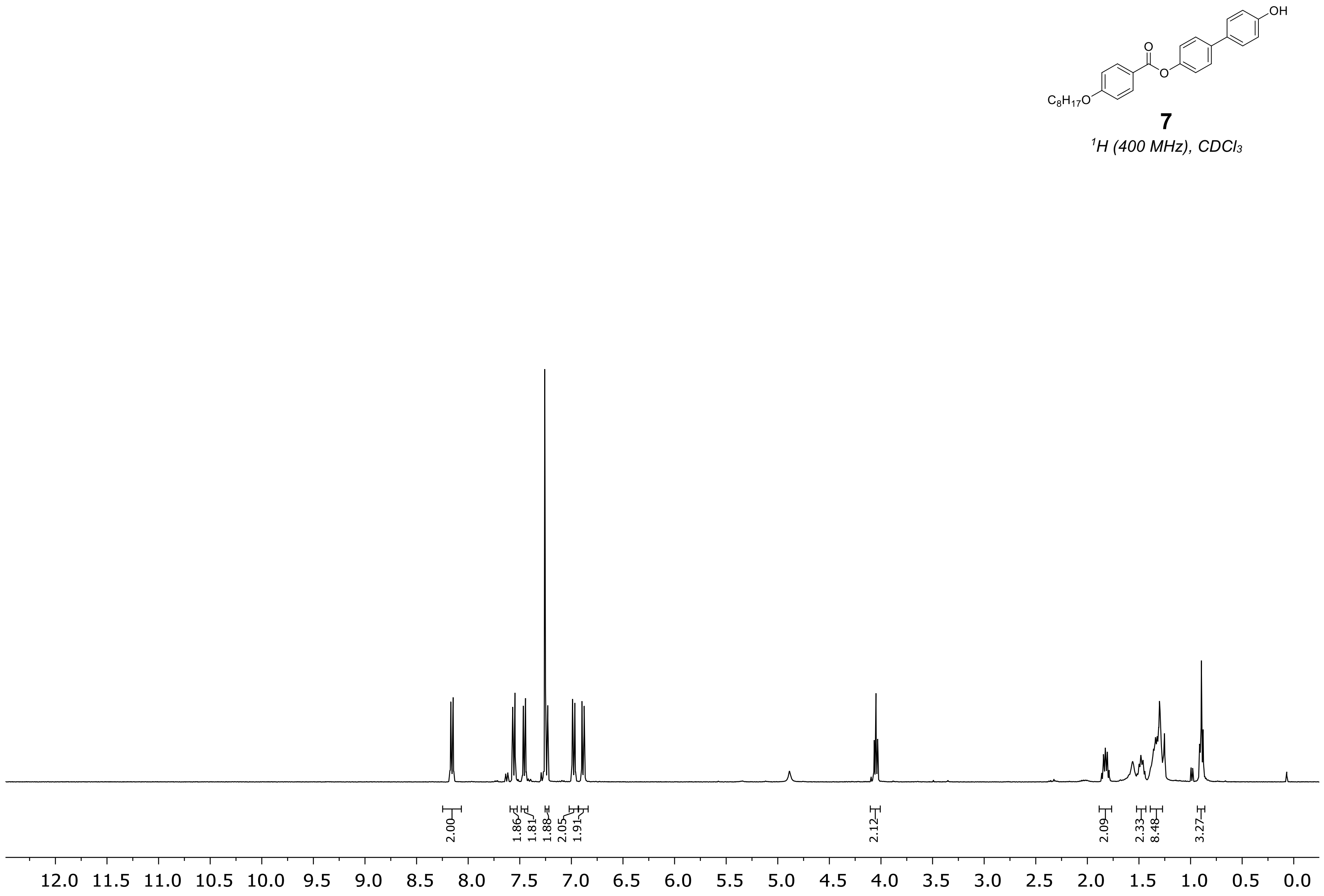

Supplementary Figure 10. ^{1}H NMR of **7**.

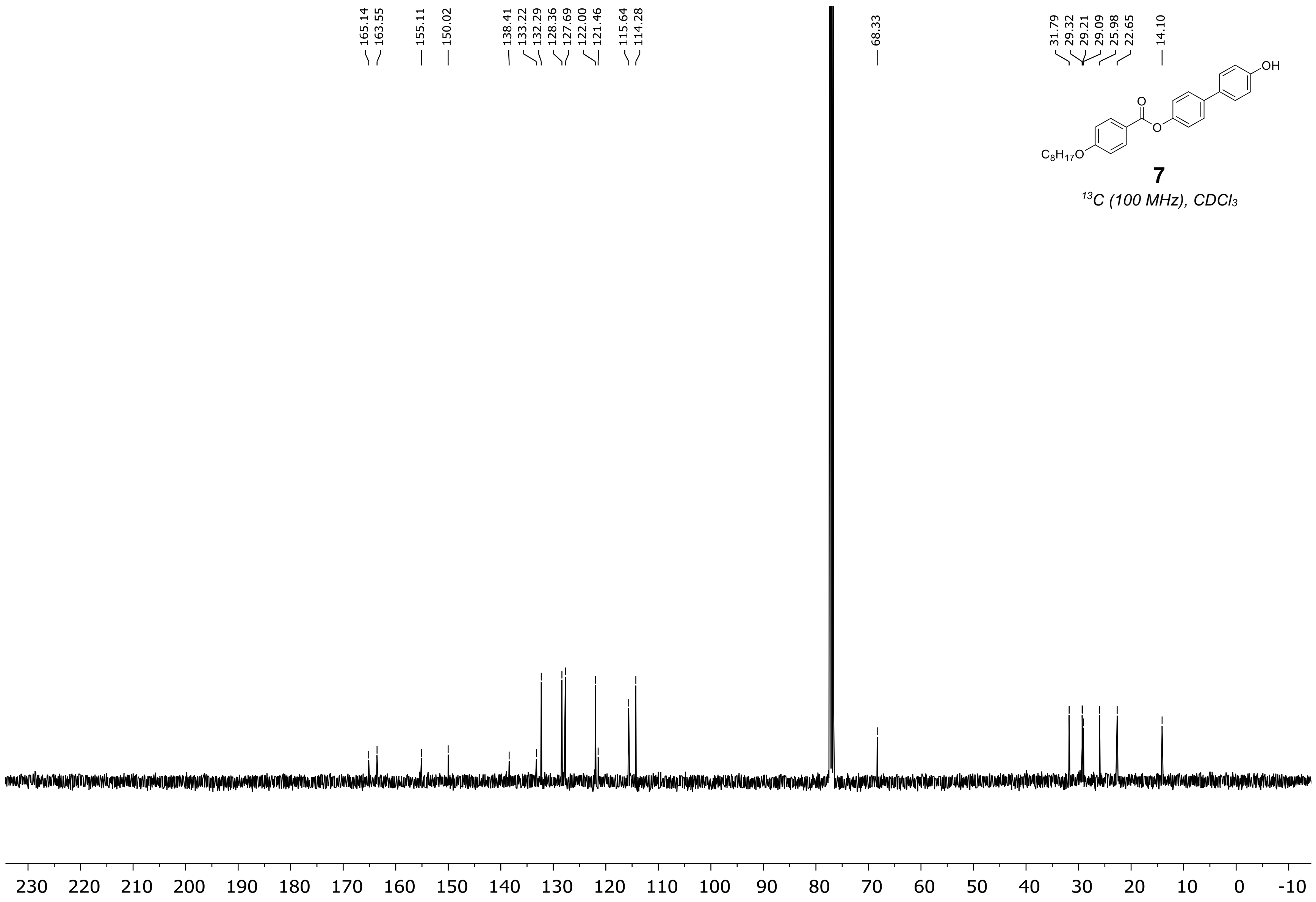

Supplementary Figure 11. ^{13}C NMR of **7**.

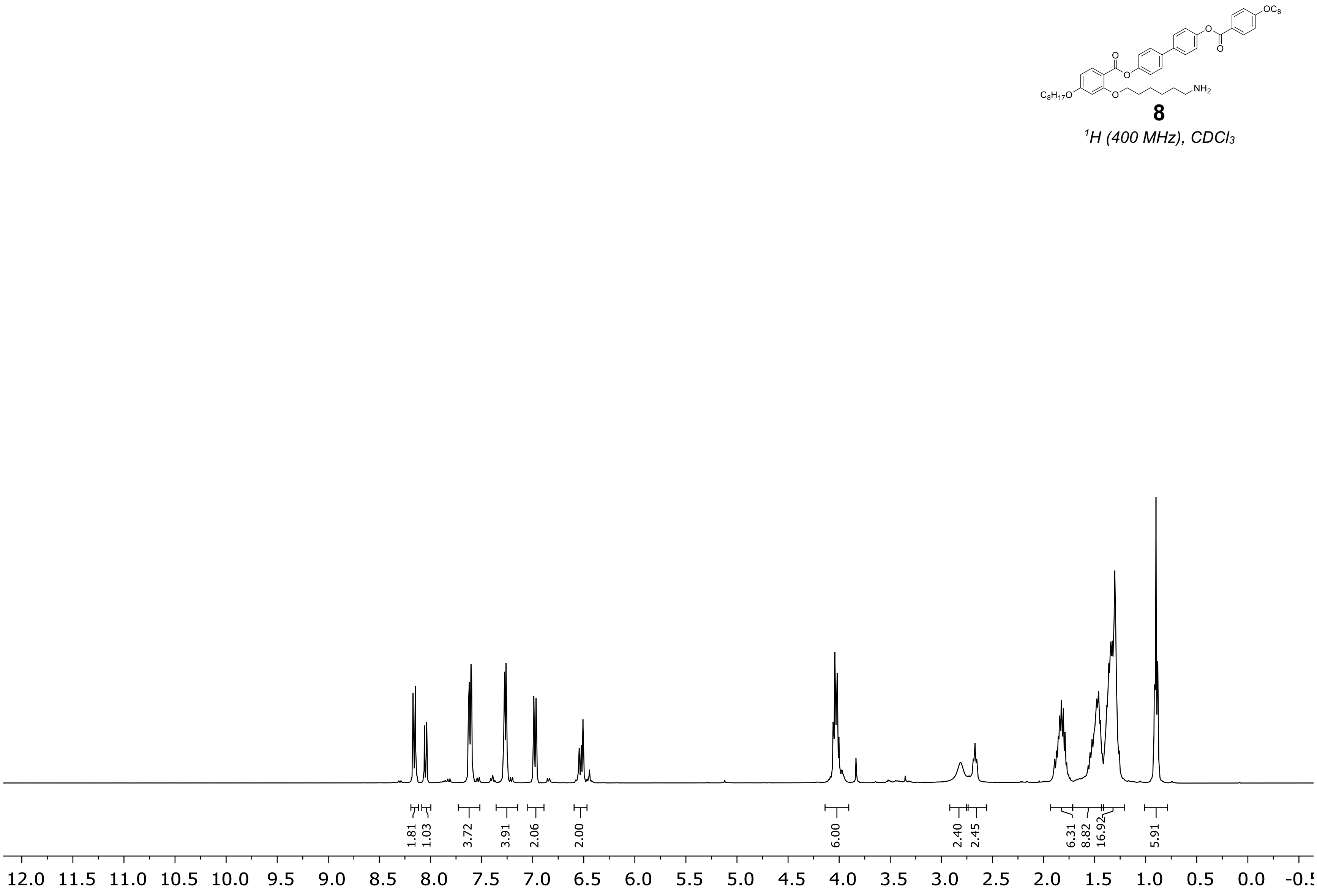

Supplementary Figure 12. ^{1}H NMR of **8**.

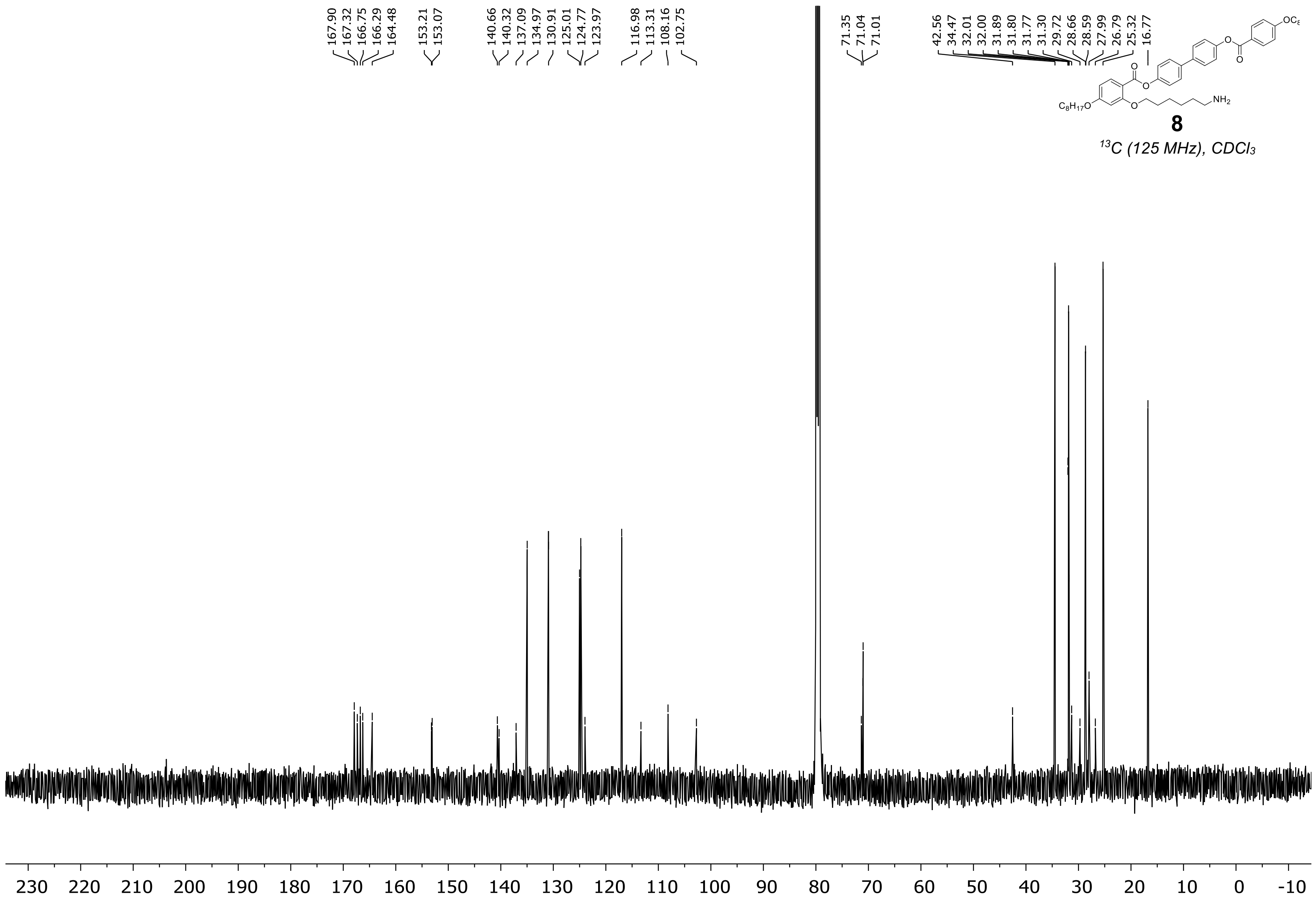

Supplementary Figure 13. ^{13}C NMR of **8**.

Supplementary References

(1) Rodarte, A. L.; Nuno, Z. S.; Cao, B. H.; Pandolfi, R. J.; Quint, M. T.; Ghosh, S.; Hein, J. E.; Hirst, L. S. *ChemPhysChem* **2014**, *15*, 1413.

(2) Quint, M. T.; Sarang, S.; Quint, D. A.; Keshavarz, A.; Stokes, B. J.; Subramaniam, A. B.; Huang, K. C.; Gopinathan, A.; Hirst, L. S.; Ghosh, S. *Sci. Rep.* **2017**, *7*, 17788.

(3) Riahinasab, S. T.; Elbaradei, A.; Keshavarz, A.; Stokes, B. J.; Hirst, L. S. *Proc. SPIE* **2017**, *10125*, 1012503–1012503-7.
