# Supplementary material for: Nanoparticle-based hollow microstructures formed by two-stage nematic nucleation and phase separation
Source: Nat Commun. 2019 Feb 22;10:894. doi: 10.1038/s41467-019-08702-3 (PMC6385213; doi:10.1038/s41467-019-08702-3)
Supplement: Supplementary file 3 — Description of Additional Supplementary Files [file 41467_2019_8702_MOESM3_ESM.pdf]

Description of Additional Supplementary Files

File Name: Supplementary Movie 1
Description: High speed fluorescence microscopy movie of hollow capsule formation. This movie focuses on few capsules in which secondary nematic domains can be seen to form inside, merging to create a hollow shell.

File Name: Supplementary Movie 2
Description: High speed fluorescence microscopy movie of hollow capsule formation. This movie focuses on the simultaneous formation of multiple capsules.
